# Long non-coding RNA HANR as a biomarker for the diagnosis and prognosis of colorectal cancer

**DOI:** 10.1097/MD.0000000000019066

**Published:** 2020-02-14

**Authors:** Meng Xu, Xu Guo, Rong-Di Wang, Zhi-Hang Zhang, Yi-Mo Jia, Xu Sun

**Affiliations:** a Department of Colorectal Surgery, Dalian Municipal Central Hospital Affiliated of Dalian Medical University; b Dalian Medical University, Dalian City, Liaoning Province, China.

**Keywords:** bio-marker, colorectal cancer, lnc-HANR, prognosis

## Abstract

Previous work suggests that the long noncoding RNA HCC associated long non-coding RNA (HANR) is associated with hepatocellular carcinoma (HCC) progression, but its significance in the context of colorectal cancer (CRC) remains to be determined. Therefore, in this study we assessed the prognostic and diagnostic value of HANR in patients suffering from CRC.

The HANR expression in 165 pairs of CRC cancer and adjacent non-cancerous prostate tissues was measured by quantitative reverse transcription polymerase chain reaction (qRT-PCR) analysis. Student *t* test was conducted for intergroup comparison. Pearson correlation test was used for correlation analysis. Survival curves were carried out by the Kaplan-Meier method and evaluated using the log-rank test. Multivariable Cox proportional hazard risk regression model was performed to screen the independent factor affected the prognosis of CRC patients.

In this study, levels of HANR were significantly higher in CRC tumor samples relative to adjacent normal tissue samples (*P* < .001). A ROC analysis suggested HANR expression could be reliably used to differentiate between normal and CRC tumor tissue. In addition, elevated HANR expression was positively correlated with more advanced and aggressive CRC features, such as a larger tumor size (*P* = .003), increased invasion depth (*P* = .012), and more advanced TNM stage (*P* = .011). Survival analyses revealed that elevated HANR expression was correlated with worse overall survival (*P* = .002) and disease-free survival (*P* = .003). A multivariate analysis further confirmed the relevance of HANR as an independent predictor of CRC patient outcomes.

In summary, these results indicate that the lncRNA HANR is a promising prognostic indicator in CRC patients.

## 1 Introduction

Colorectal cancer (CRC) remains the second deadliest cancer affecting males, resulting in greater than 600,000 deaths each year globally.^[1,2]^ CRC arises over time with a successive series of histological and genetic alterations in the underlying tissue.^[3]^ Those patients with stage I/II CRC have a generally good prognosis owing to recent advances in targeted, surgical, and chemotherapeutic treatments, with a 5-year survival rate of up to 80%.^[4,5]^ Over half of CRC patients, however, exhibit distant metastases at diagnosis, and limited therapeutic options are available for the treatment of such advanced disease, with very complex therapy being essential.^[6,7]^ As such it is important that new biomarkers of diagnostic or prognostic utility be identified in an effort to improve patient survival rates.

Long noncoding RNAs (lncRNAs) are RNA molecules that do not encode protein despite their active transcription.^[8]^ There is clear evidence^[9,10]^ that certain lncRNAs are able to act through a variety of mechanisms in order to influence diverse processes including transcription, mRNA stability, and epigenetic regulatory pathways. In addition, several studies^[11,12]^ have found that certain lncRNAs are dysregulated in tumors, with those being overexpressed often helping to initiate or drive tumor development. As an example, UCC is a lncRNA found to be overexpressed in certain CRC samples, and to be capable of enhancing the metastatic and proliferative activity of CRC cells via sequestering miR-143, with elevated expression of this lncRNA being correlated with more advanced disease.^[13]^ In contrast, Linc00675 has been shown to be downregulated in CRC patients, and in these cells it has been found to act to suppress cellular invasion and proliferation through regulation of Wnt/β-catenin signaling.^[14]^ These results thus demonstrate that in CRC lncRNAs have potential and meaningful prognostic and diagnostic biomarkers.

HCC-associated lncRNA (HCC associated long non-coding RNA [HANR]; RPL13AP20)^[15]^ has been shown to drive the enhanced proliferation, invasion, and epithelial-mesenchymal transition (EMT) of certain cancer cells.^[16,17]^ Whether HANR exhibits any clinical relevance in patients with CRC, however, is unclear. As such, this study sought to produce novel evidence examining the potential for HANR to be a diagnostic and/or prognostic biomarker in CRC patients.

## 2 Patients and methods

### 2.1 Patients and clinical specimens

A total of 165 pairs of CRC tumor tissue and adjacent healthy tissue were collected by Dalian Central Hospital. These patients (101 males, 64 female) were 38 to 76 years old (median: 53.9 years), and had been diagnosed with CRC based upon both clinical and histopathological assessments. Samples used in this study were from patients who had not undergone preoperative chemotherapy or radiotherapy. Samples were snap-frozen using liquid nitrogen prior to −80°C storage. Patient follow-up information and survival was determined based upon medical records and or direct contact with the patients or their families. Patient demographic and clinical characteristics are compiled in Table 1.

**Table 1:**
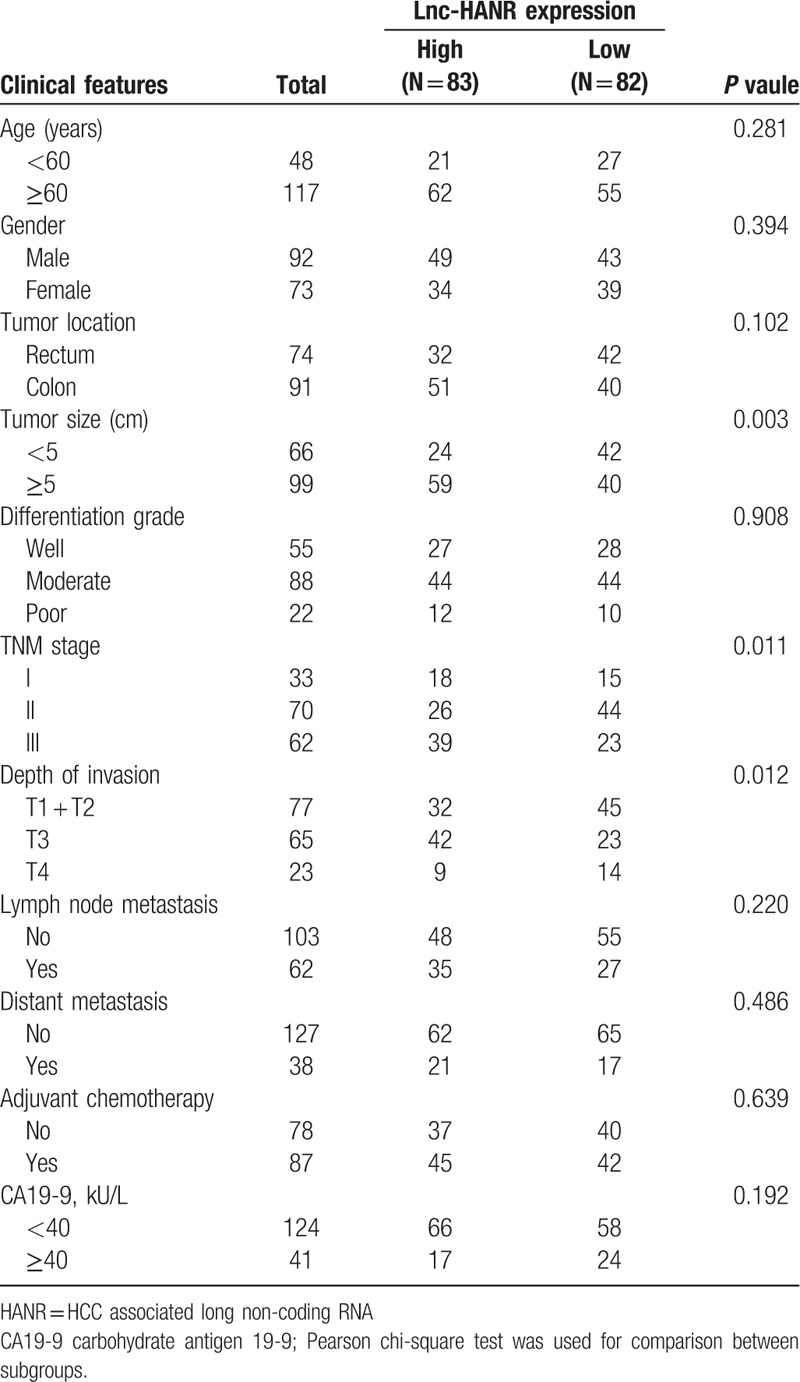
Association between Lnc-HANR expression and clinicopathological features of human CRC.

The approval of the present study protocol was obtained from the Ethics Committee of the Dalian Central Hospital (Affiliated of Dalian Medical University), and the written informed consent was provided from all patients.

### 2.2 qRT-PCR

RNA was extracted from samples using the miRNeasy Mini-kit (Qiagen, Xuhui, Shanghai, China), after which Reverse Transcriptase (Transgene, Xuhui, Shanghai, China) was used to prepare cDNA from all samples. A 7300-sequence detection system (Biosystems, CA) was used to conduct qRT-PCR reactions with SYBR Green Master Mix (Biosystems) using 35 cycles of 12 seconds at 95°C and 1 minute at 60°C. For normalization, GAPDH was used as an endogenous control gene. The comparative cycle threshold (CT) approach was used for determining relative HANR expression levels, with primers used shown in Table 2.

**Table 2:**
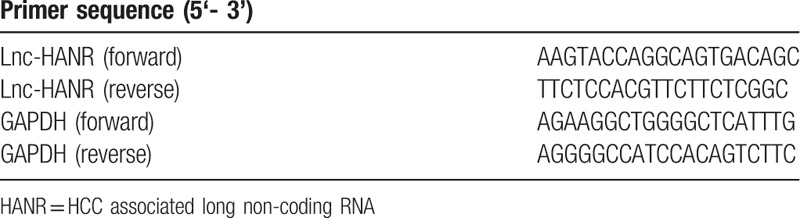
The primers used for qRT-PCR analysis.

### 2.3 Statistical analysis

SPSS 17.0 (SPSS Inc, IL) was used for statistical testing. Student's *t* tests were used to compare data between groups, while the association between HANR expression and clinicopathological findings was assessed via chi-squared test. A receiver operating characteristic (ROC) curve analysis was used to assess the diagnostic relevance of HANR expression in CRC patients, while Kaplan-Meier and multivariate analyses were used to gauge the prognostic value of HANR expression. *P* < .05 was the significance threshold.

## 3 Results

### 3.1 CRC tumors exhibit elevated HANR expression

**Figure 1:**
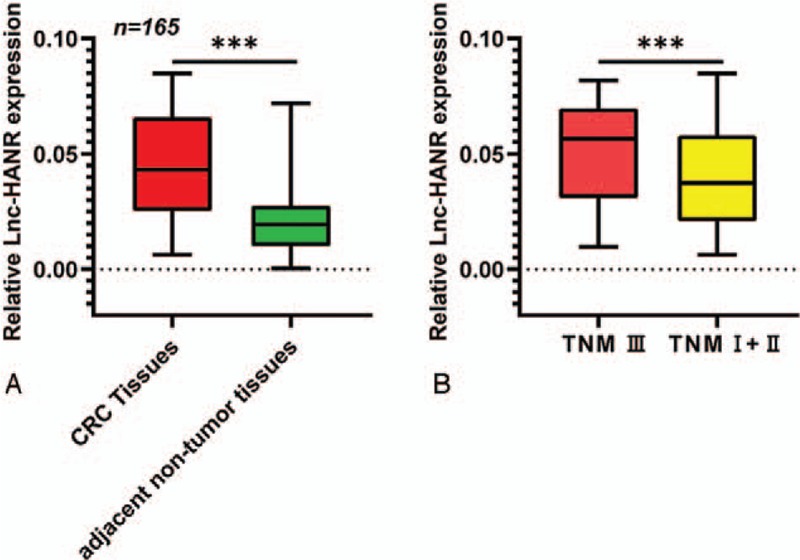
CRC tumors exhibit decreased HANR expression. (A) qRT-PCR was used to assess HANR expression in 165 paired normal and CRC tumor tissues. (B) CRC patients with more advanced disease exhibited higher levels of HANR expression. CRC = colorectal cancer, HANR = HCC associated long non-coding RNA.

To first assess how the lncRNA HANR might be linked to CRC progression, we explored its expression levels in tumor samples from those patients suffering from CRC. This analysis revealed that tumor samples exhibited significantly higher HANR expression relative to normal control samples (*P* < .01) (Fig. 1A). In addition, patients with more advanced CRC exhibited significantly higher HANR expression than those with less advanced disease (*P* < .01, Fig. 1B). Together these findings suggested the possibility that the lncRNA HANR is expressed at high levels in CRC and may play a role in disease progression.

### 3.2 The diagnostic value of HANR expression in CRC patients

**Figure 2:**
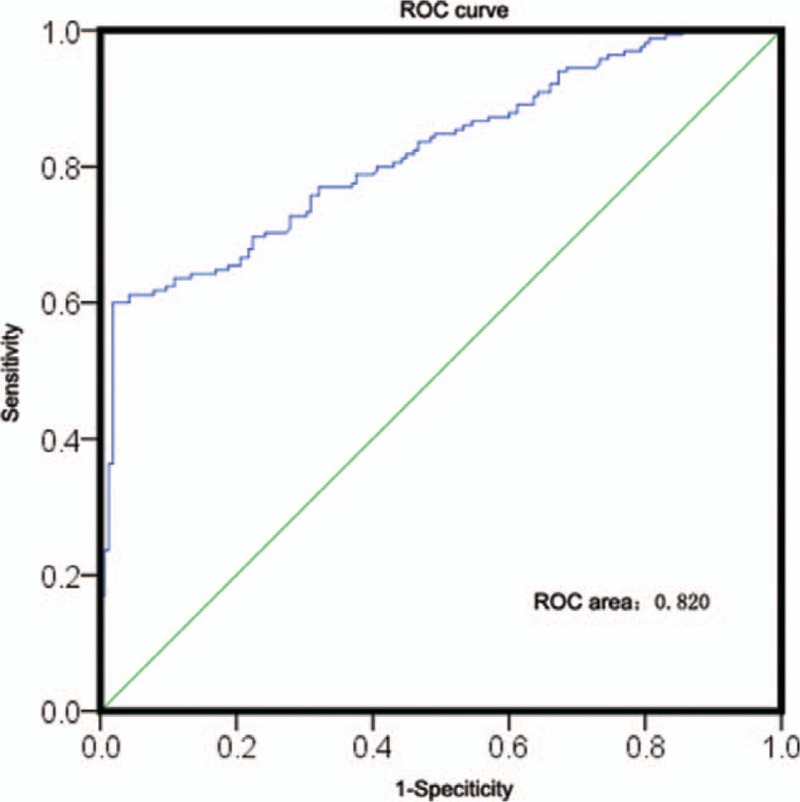
Receiver operating characteristic curve analysis of the diagnostic value of the lncRNA HANR in colorectal cancer. HANR = HCC associated long non-coding RNA

As HANR expression was apparently dysregulated in CRC patient samples, we next explored its potential utility as a diagnostic biomarker of CRC. A ROC curve analysis suggests that HANR expression levels allowed for reliable differentiation between normal and CRC tumor tissues (AUC: 0.820; 95% confidence interval: 0.775–0.865) (Fig. 2). The sensitivity and specificity of HANR in this analysis were 0.60 and 0.82, respectively. The lncRNA HANR may thus be a useful diagnostic biomarker for CRC.

### 3.3 Elevated HANR expression correlated with CRC patient clinical characteristics

To further assess the clinical relevance of HANR expression in patients with CRC, we divided the 165 patient samples according to their levels of HANR expression (HANR-high or HANR-low; n = 83 and 82, respectively) based on the median HANR expression level in CRC tumor tissue samples. Chi-squared tests were then used to compare clinical characteristics between groups, revealing that higher HANR levels were associated with tumor size (*P* = .003), depth of invasion (*P* = .012), and advanced TNM stage (*P* = .011) (Table 1). In contrast, there was no relationship between HANR expression and patient age, gender, histological findings, or tumor site (*P* > .05).

### 3.4 HANR offers prognostic utility in CRC patients

**Figure 3:**
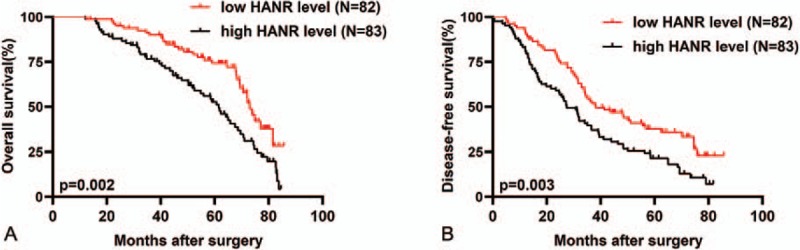
CRC patient survival is associated with HANR expression levels. (A) CRC patients with high HANR expression exhibited significantly reduced overall survival relative to HANR-high patients (*P* = .002). (B) CRC patients with high HANR expression exhibited significantly reduced disease-free survival relative to HANR-high patients (*P* = .003). CRC = colorectal cancer, HANR = HCC associated long non-coding RNA.

Finally, we assessed the prognostic relevance of HANR in CRC via a Kaplan-Meier approach, revealing a significant association between elevated HANR expression and reduced overall survival (OS) (*P* = .002, Fig. 3A) as well as disease-free survival (DFS) (*P* = .003, Fig. 3B), meaning that higher levels of this lncRNA are correlated with a worse prognosis. A multivariate analysis was additionally conducted to identify factors predictive of OS and DFS (Table 3), revealing that elevated HANR expression independently predicted reduced OS (HR = 2.501, 95% CI: 1.956–4.108, *P* = .023) and DFS (HR = 2.314, 95% CI: 1.713–3.956, *P* = .012) in CRC patients.

**Table 3:**
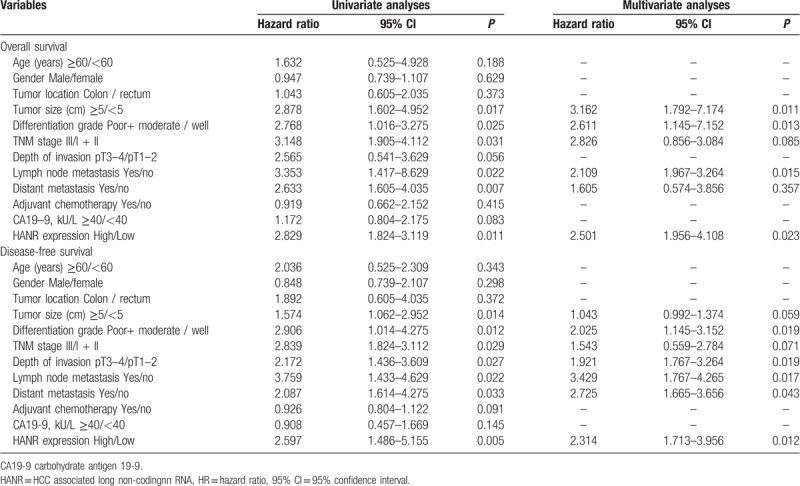
Univariate and multivariate Cox regression analyses of overall survival and disease-free survival in CRC patients.

## 4 Discussion

CRC is a form of cancer that remains highly aggressive, with many patients succumbing to the disease, making it the 5th leading cause of cancer-associated death in China.^[18]^ Ongoing research has highlighted new avenues for cancer detection and treatment, with specific biomarkers having great promise for the detection and management of CRC.^[19,20]^ Current factors used to guide patient treatment include KRAS sequencing and measures of micro-satellite instability.^[21]^ Several studies^[22,23]^ have highlighted the potential of lncRNAs to serve as tumor diagnostic biomarkers, given that these RNA molecules are often dysregulated in tumors in a manner functionally linked to tumor progression. As high throughput sequencing technologies have become increasingly prevalent, it has become far easier to readily detect expression patterns of many lncRNAs at the same time, making them ideal targets worth of study as putative diagnostic and prognostic biomarkers.^[24,25]^

Many reports have sought to characterize patterns of lncRNA expression and their functional relevance in CRC.^[26]^ For example, Han et al^[27]^ found lncRNA H19 to be expressed at high levels in CRC in a manner correlated with reduced CRC patient survival and enhanced tumor growth owing to its ability to bind eIF4A3. Iguchi et al^[28]^ found lncRNA-ATB to be expressed at high levels in CRC and to correspond with a worse patient prognosis. Zhang et al^[29]^ found the lncRNA HNF1A-AS1 to similarly be overexpressed in CRC patients in a manner correlating with poorer survival, with in vitro analyses demonstrating the ability of this lncRNA to regulate Wnt/β-catenin signaling so as to control tumor cell invasion. HANR was recently shown to be overexpressed in HCC,^[15–17]^ leading us to investigate its relevance in CRC. Xiao et al^[15]^ firstly observed increased HANR levels in HCC patient tissues and cells, with higher levels of this lncRNA corresponding to poorer survival. When HANR was knocked down, cell proliferation and tumor growth in vivo was impaired, with tumors becoming more sensitive to chemotherapeutic treatment. In contrast, overexpression of this lncRNA had the opposite effect. The authors determined HANR to be capable of binding GSKIP, thereby controlling GSK3β phosphorylation in HCC, potentially thereby promoting tumor growth. Whether HANR is similarly relevant to the prognosis of CRC patients has not been assessed previously.

In line with previous findings, we observed a significant upregulation of HANR in CRC tumors relative to adjacent normal controls, with ROC curve analyses confirming that HANR may be an effective marker well-suited to differentiating between normal and tumor tissue. We further provided novel insight into the clinical relevance of HANR, determining that it was significantly associated with tumor size, depth of invasion, and more advanced TNM stage, indicating that HANR may be positively associated with CRC progression in patients. Importantly, when we assessed patient survival as a function of HANR expression we found that individuals with higher HANR expression suffered poorer clinical outcomes, with shorter average OS and DFS. We then employed a multivariate analysis to demonstrate that HANR was an independent predictor of OS and DFS in CRC patients, confirming its potential relevance. The limitation of the present study is that:

(1) we have not investigated the targets of onco-HANR such as miR-214, miR-296, or EAG1 in CRC cells. More in-depth study is needed in the future to clarify the role of HANR in CRC.
(2) we employed an arbitrary HANR expression level cut-off value in this study, and future research should seek to identify an optimal clinically relevant cut-off value.
(3) In addition, our study population was relatively small, and as such additional research will be needed to confirm that HANR is relevant as a biomarker in CRC patients.

In summary, these results indicate that HANR has potential as a novel biomarker useful for diagnosing CRC and/or for predicting patient prognosis, with higher levels of this lncRNA being correlated with poorer patient prognosis.

## Author contributions

]

**Conceptualization:** Meng Xu.

**Data curation:** Meng Xu, Zhi-hang Zhang.

**Funding acquisition:** Yi-Mo Jia, Xu Sun.

**Investigation:** Zhi-hang Zhang, Xu Sun.

**Methodology:** Zhi-hang Zhang, Yi-Mo Jia, Xu Sun.

**Writing – original draft:** Meng Xu, Xu Guo, Rong-Di Wang, Yi-Mo Jia, Xu Sun.

**Writing – review & editing:** Yi-Mo Jia.

## References

[1] Siegel RL Miller KD Jemal A Cancer statistics, 2017. CA Cancer J Clin 2017;67:7–30. 28055103 10.3322/caac.21387

[2] Siegel RL Miller KD Fedewa SA Colorectal cancer statistics, 2017. CA Cancer J Clin 2017;67:177–93. 28248415 10.3322/caac.21395

[3] Stoffel EM Yurgelun MB Genetic predisposition to colorectal cancer: Implications for treatment and prevention. Semin Oncol 2016;43:536–42. 27899184 10.1053/j.seminoncol.2016.08.002

[4] Kim JH Chemotherapy for colorectal cancer in the elderly. World J Gastroenterol 2015;21:5158–66. 25954089 10.3748/wjg.v21.i17.5158 PMC4419056

[5] Brenner H Kloor M Pox CP Colorectal cancer. Lancet 2014;383:1490–502. 24225001 10.1016/S0140-6736(13)61649-9

[6] Xiang B Snook AE Magee MS Colorectal cancer immunotherapy. Discov Med 2013;15:301–8. 23725603 PMC4042089

[7] Kennedy AS The role of radioembolization in colorectal cancer treatment. Expert Rev Anticancer Ther 2016;16:375–6. 26938710 10.1586/14737140.2016.1161509

[8] Marchese FP Raimondi I Huarte M The multidimensional mechanisms of long noncoding RNA function. Genome Biol 2017;18:206. 29084573 10.1186/s13059-017-1348-2 PMC5663108

[9] Kung JT Colognori D Lee JT Long noncoding RNAs: past, present, and future. Genetics 2013;193:651–69. 23463798 10.1534/genetics.112.146704 PMC3583990

[10] Askarian-Amiri ME Leung E Finlay G The regulatory role of long noncoding RNAs in cancer drug resistance. Methods Mol Biol 2016;1395:207–27. 26910076 10.1007/978-1-4939-3347-1_12

[11] Schmitt AM Chang HY Long noncoding RNAs: at the intersection of cancer and chromatin biology. Cold Spring Harb Perspect Med 2017;7:a026492. 28193769 10.1101/cshperspect.a026492 PMC5495049

[12] Yang X Duan B Zhou X Long non-coding RNA FOXD2-AS1 functions as a tumor promoter in colorectal cancer by regulating EMT and Notch signaling pathway. Eur Rev Med Pharmacol Sci 2017;21:3586–91. 28925486

[13] Huang FT Chen WY Gu ZQ The novel long intergenic noncoding RNA UCC promotes colorectal cancer progression by sponging miR-143. Cell Death Dis 2017;8:e2778. 28492554 10.1038/cddis.2017.191 PMC5520712

[14] Shan Z An N Qin J Long non-coding RNA Linc00675 suppresses cell proliferation and metastasis in colorectal cancer via acting on miR-942 and Wnt/(-catenin signaling. Biomed Pharmacother 2018;101:769–76. 29524886 10.1016/j.biopha.2018.02.123

[15] Xiao J Lv Y Jin F LncRNA HANR promotes tumorigenesis and increase of chemoresistance in hepatocellular carcinoma. Cell Physiol Biochem 2017;43:1926–38. 29055955 10.1159/000484116

[16] Shi Y Yang X Xue X HANR promotes hepatocellular carcinoma progression via miR-214/EZH2/TGF-( axis. Biochem Biophys Res Commun 2018;506:189–93. 30342849 10.1016/j.bbrc.2018.10.038

[17] Shi Y Yang X Xue X HANR promotes lymphangiogenesis of hepatocellular carcinoma via secreting miR-296 exosome and regulating EAG1/VEGFA signaling in HDLEC cells. J Cell Biochem 2019;120:17699–708. 31127654 10.1002/jcb.29036

[18] Zhu J Tan Z Hollis-Hansen K Epidemiological trends in colorectal cancer in china: an ecological study. Dig Dis Sci 2017;62:235–43. 27796769 10.1007/s10620-016-4362-4

[19] Aghagolzadeh P Radpour R New trends in molecular and cellular biomarker discovery for colorectal cancer. World J Gastroenterol 2016;22:5678–93. 27433083 10.3748/wjg.v22.i25.5678 PMC4932205

[20] Schirripa M Lenz HJ Biomarker in colorectal cancer. Cancer J 2016;22:156–64. 27341592 10.1097/PPO.0000000000000190 PMC4955946

[21] Binefa G Rodríguez-Moranta F Teule A Colorectal cancer: from prevention to personalized medicine. World J Gastroenterol 2014;20:6786–808. 24944469 10.3748/wjg.v20.i22.6786 PMC4051918

[22] Yang JP Yang XJ Xiao L Long noncoding RNA PVT1 as a novel serum biomarker for detection of cervical cancer. Eur Rev Med Pharmacol Sci 2016;20:3980–6. 27775803

[23] Mohankumar S Patel T Extracellular vesicle long noncoding RNA as potential biomarkers of liver cancer. Brief Funct Genomics 2016;15:249–56. 26634812 10.1093/bfgp/elv058 PMC4880007

[24] Ferrè F Colantoni A Helmer-Citterich M Revealing protein-lncRNA interaction. Brief Bioinform 2016;17:106–16. 26041786 10.1093/bib/bbv031 PMC4719072

[25] Lagarde J Uszczynska-Ratajczak B Carbonell S High-throughput annotation of full-length long noncoding RNAs with capture long-read sequencing. Nat Genet 2017;49:1731–40. 29106417 10.1038/ng.3988 PMC5709232

[26] Zheng Q Lin Y Chen P The long noncoding RNA BC0209135 inhibits the cell invasion through Wnt/(-catenin signaling in colorectal cancer. Eur Rev Med Pharmacol Sci 2018;22:3763–70. 29949151 10.26355/eurrev_201806_15258

[27] Han D Gao X Wang M Long noncoding RNA H19 indicates a poor prognosis of colorectal cancer and promotes tumor growth by recruiting and binding to eIF4A3. Oncotarget 2016;7:22159–73. 26989025 10.18632/oncotarget.8063 PMC5008352

[28] Iguchi T Uchi R Nambara S A long noncoding RNA, lncRNA-ATB, is involved in the progression and prognosis of colorectal cancer. Anticancer Res 2015;35:1385–8. 25750289

[29] Zhang X Xiong Y Tang F Long noncoding RNA HNF1A-AS1 indicates a poor prognosis of colorectal cancer and promotes carcinogenesis via activation of the Wnt/β-catenin signaling pathway. Biomed Pharmacother 2017;96:877–83. 29145164 10.1016/j.biopha.2017.10.033

